# Analysis of the interaction of monoclonal antibodies with surface IgM on neoplastic B-cells

**DOI:** 10.1038/sj.bjc.6690136

**Published:** 1999-02

**Authors:** M S Cragg, L Zhang, R R French, M J Glennie

**Affiliations:** Tenovus Research Laboratory, Southampton General Hospital, Tremona Road, Southampton, SO16 6YD, UK; Medical Research Laboratory of Molecular Biology, Hills Road, Cambridge, CB2 2QH, UK

**Keywords:** monoclonal antibody, transmembrane signalling, surface IgM, crosslinking, antibody immunotherapy

## Abstract

In vitro studies identified three Burkitts lymphoma cell lines, Ramos, MUTU-I and Daudi, that were growth inhibited by anti-IgM antibody. However, only Ramos and MUTU-I were sensitive to monoclonal antibodies (mAb) recognizing the Fc region of surface IgM (anti-Fcμ). Experiments using anti-Fcμ mAb (single or non-crossblocking pairs), polyclonal anti-μ Ab, and hyper-crosslinking with a secondary layer of Ab, showed that growth inhibition of B-cell lines was highly dependent on the extent of IgM crosslinking. This was confirmed by using Fab′, F(ab′)_2_and F(ab′)_3_derivatives from anti-Fcμ mAb, where increasing valency caused corresponding increases in growth arrest and apoptosis, presumably as a result of more efficient BCR-crosslinking on the cell surface. The ability of a single mAb to induce growth arrest was highly dependent on epitope specificity, with mAb specific for the Fc region (Cμ2–Cμ4 domains) being much more effective than those recognizing the Fab region (anti-L chain, anti-Id and anti-Fdμ, or Cμ1). Only when hyper-crosslinked with polyclonal anti-mouse IgG did the latter result in appreciable growth inhibition. Binding studies showed that these differences in function were not related to differences in the affinity, but probably related to intrinsic crosslinking capacity of mAb. © 1999 Cancer Research Campaign


*British Journal of Cancer* (1999) **79**(5/6), 850–857
© 1999 Cancer Research Campaign
Article no. bjoc.1998.0136

### Analysis of the interaction of monoclonal antibodies with surface IgM on neoplastic B-cells

**MS Cragg[1], L Zhang[2], RR French[1] and MJ Glennie[1]**

[1]Tenovus Research Laboratory, Southampton General Hospital, Tremona Road, Southampton, SO16 6YD, UK; [2]Medical Research Laboratory of Molecular Biology, Hills Road, Cambridge, CB2 2QH, UK

**Summary** In vitro studies identified three Burkitts lymphoma cell lines, Ramos, MUTU-I and Daudi, that were growth inhibited by anti-IgM antibody. However, only Ramos and MUTU-I were sensitive to monoclonal antibodies (mAb) recognizing the Fc region of surface IgM (anti-Fcμ). Experiments using anti-Fcμ mAb (single or non-crossblocking pairs), polyclonal anti-μ Ab, and hyper-crosslinking with a secondary layer of Ab, showed that growth inhibition of B-cell lines was highly dependent on the extent of IgM crosslinking. This was confirmed by using Fab′, F(ab′)$_2$ and F(ab′)$_3$ derivatives from anti-Fcμ mAb, where increasing valency caused corresponding increases in growth arrest and apoptosis, presumably as a result of more efficient BCR-crosslinking on the cell surface. The ability of a single mAb to induce growth arrest was highly dependent on epitope specificity, with mAb specific for the Fc region (Cμ2–Cμ4 domains) being much more effective than those recognizing the Fab region (anti-L chain, anti-Id and anti-Fdμ, or Cμ1). Only when hyper-crosslinked with polyclonal anti-mouse IgG did the latter result in appreciable growth inhibition. Binding studies showed that these differences in function were not related to differences in the affinity, but probably related to intrinsic crosslinking capacity of mAb.

**Keyword:** monoclonal antibody; transmembrane signalling; surface IgM; crosslinking; antibody immunotherapy

In vitro studies have shown that crosslinking of the B-cell receptor (BCR) by anti-IgM monoclonal antibody (mAb) can have either stimulatory or inhibitory effects depending on the maturation state of the cell (De Franco et al, 1982; Hasbold and Klaus, 1990.). Of particular interest in the field of immunotherapy is that crosslinking of the BCR on B lymphoma cells can induce inhibitory signals that may result in cell cycle arrest and apoptosis (Hasbold and Klaus, 1990; Marches et al, 1995). There is growing evidence that such transmembrane signalling is critical to much of the therapeutic success of anti-idiotype (Id) mAb in the treatment of non-Hodgkins lymphoma (NHL). For example the study by Vuist et al (1994) highlighted the close correlation between the therapeutic success of anti-Id mAb in NHL and the ability of the treatment mAb to stimulate protein tyrosine phosphorylation in frozen archive tumour material. Since tyrosine phosphorylation following crosslinking of surface immunoglobulin (Ig) is one of the first steps in the cascade of intracellular signalling events (Gold et al, 1991; Sefton and Campbell, 1991), this positive correlation suggests that the key to the success of anti-Id mAb may stem from their ability to crosslink surface Ig and deliver a direct anti-proliferative signal to the tumour. The importance of crosslinking was further suggested by the observation that although cells from non-responding patients could not be induced to signal with anti-Id mAb, they were responsive to stimulation with a polyclonal anti-μ Ab, which, because it recognizes multiple epitopes on the BCR, possesses a far greater potential for crosslinking than a single mAb.

While these results indicate the importance of crosslinking in the inhibitory action of anti-Id mAb, the extent of crosslinking necessary for achieving inhibition, and the antibody binding requirements, have not been fully defined. In this paper, we describe the sensitivity of a panel of Burkitt's lymphoma cell lines to the growth inhibitory effects of anti-IgM mAb, and a series of experiments designed to look at the effects of IgM domain specificity, BCR crosslinking, and hyper-crosslinking on growth inhibition.

*Received 8 June 1998*
*Revised 5 August 1998*
*Accepted 7 August 1998*

*Correspondence to:* MS Cragg

### MATERIALS AND METHODS

### Cell lines and antibodies

The Burkitt's lymphoma cell lines Daudi, Ramos, Ramos-sublines RA.1 and EHRB, Raji, Namalwa, MUTU-I and MUTU-61E (kindly given by Dr A Milner and Prof. Chris Gregory, Birmingham, UK), Nalm-6 (kindly given by Dr Bill Cushley, Glasgow, UK) and Bristol-8 cells were used in this study. All cell lines were maintained in supplemented RPMI-1640 [RPMI-1640 medium containing glutamine (2 mM), pyruvate (1 mM), penicillin and streptomycin (100 IU ml$^{-1}$), fungizone (2 μg ml$^{-1}$) and 10% fetal calf serum (FCS) (Myoclone) (Gibco Ltd, Paisley, UK)]. Monoclonal antibodies used in these studies included: anti-μ, M15/8, ZL7/5 and Mc41/24G10 (all anti-Fcμ), and anti-λ, M15/2 and Mc24/1C6, all raised in this laboratory (Bonardi et al, 1993; Zhang et al, 1995), the anti-Fabμ antibodies M-2E6 (ATCC, Rockville, MA, USA) and XG9 (kindly given by Dr Patricia Mongini, New York, NY, USA) (Rudich et al, 1988). In addition, we used three anti-Id mAb, ZL16/1, ZL16/3 and ZL16/11, specific for the surface IgM of the Ramos cell line and one anti-Id mAb, ZL15/27, specific for the surface IgM of the Daudi cell line (Zhang et al, 1995). Polyclonal sheep anti-mouse IgG was raised at Tenovus.

All hybridoma lines secreting mAb were expanded in tissue culture and purified from culture supernatant using a Protein A column (Glennie et al, 1993). F(ab′)$_2$ fragments of IgG were prepared by limited proteolysis with pepsin at pH 4.0–4.2 (Glennie et al, 1993). Fab′ from M15/8 and Mc24/1C6 F(ab′)$_2$ was produced by reduction with 20 mM 2-mercaptoethanol for 30 min at 25°C, followed by alkylation with excess iodoacetamide.

### Measurement of [$^3$H]thymidine incorporation

Cells (5 × 10$^4$) were aliquoted into each well of a 96-well microtitre plate (Gibco) and incubated in 200 μl of supplemented RPMI medium containing the required concentration of mAb for 24 h at 37°C before pulsing overnight with 0.5 μCi [$^3$H]thymidine (Amersham International PLC, UK) in 50 μl RPMI as described previously (Tutt et al, 1991). The incorporation of [$^3$H]thymidine into DNA was assessed by harvesting the cells onto glass microfibre filters and washing with water. Experimental points were determined in triplicate. The variation between triplicates was less than 10%.

### Preparation of anti-Fcμ (M15/8) multimers

M15/8 F(ab′)$_3$ was prepared using the bis-maleimide crosslinker, *o*-phenylenedimaleimide, as described by Tutt et al (1991) for the preparation of bispecific trimers.

### Hyper-crosslinking experiments mAb at a concentration of 10 μg ml$^{-1}$ were first allowed to bind to cells at a concentration of 2.5 × 10$^5$ ml$^{-1}$ for 15 min at 4°C. Cells were then separated from excess unbound antibody in the supernatant by centrifugation, and the supernatant removed. Cells were resuspended at a concentration of 2.5 × 10$^5$ ml$^{-1}$ in medium containing polyclonal sheep anti-mouse IgG at 20 μg ml$^{-1}$, to act as a hyper-crosslinking reagent, plated out as 200 μl aliquots on 96-well microtitre plates, and incubated for 24 h at 37°C before pulsing overnight with 0.5 μCi [$^3$H]thymidine. Cells were then harvested and incorporated radioactivity determined as described above.

### Measurement of apoptosis using propidium iodide

Samples were assessed by the method of Nicoletti et al (1991). Briefly, aliquots of cells (10$^6$) were centrifuged for 5 min at 500 ***g*** and then washed once in phosphate-buffered saline (PBS). The cells were then resuspended in hypotonic fluorochrome solution (50 μg ml$^{-1}$ propidium iodide (PI), 0.1% (w/v) sodium citrate, 0.1% (w/v) Triton X-100) and incubated in the dark at 4°C overnight or 1 h at room temperature. PI staining of DNA from 10 000 cells was detected on a FACScan flow cytometer (Becton Dickinson) and the proportion of DNA giving fluorescence below the G1/0 peak was taken as a measure of apoptosis.

### Agarose gel DNA fragmentation analysis

Following incubation of cells under appropriate conditions, samples containing 1–2 × 10$^6$ cells were harvested. Twenty microlitres TE-SLS (10 mM EDTA, 50 mM Tris-HCl pH 8.0, 0.5% (v/v) sodium lauryl sarkosinate (SLS, Sigma)) plus 0.5 mg ml$^{-1}$ proteinase K (Sigma) was then added, and the samples incubated for 1–2 h at 50°C. 10 μl 0.16 mg ml$^{-1}$ RNase A (Sigma) was added and samples were incubated for a further 1–2 h at 50°C. The temperature of the samples was then increased to 70°C for addition of 10 μl of loading buffer [10 mM EDTA, 1% (w/v) low melting point agarose, 0.25% (w/v) bromophenol blue, 40% (w/v) sucrose]. Samples were loaded into dry wells of 1% (w/v) agarose gels containing 0.5 mg ml$^{-1}$ ethidium bromide and run either overnight at 30–40 V or for 2–3 h at 80 V, in TAE (89 mM Tris, 89 mM boric acid, 2 mM EDTA, pH8.4). DNA was visualized by UV light.

### Radioiodination of antibodies mAb were trace radiolabelled for binding studies using carrier-free $^{125}$I (Amersham International plc) and IODO-BEADS (Pierce Chemicals Co., Rockford, IL, USA) as the oxidizing reagent. Radioactivity was measured in a Rackgamma spectrometer (LKB). The immunoreactive component of each mAb [$^{125}$I]IgG preparation was estimated by determining what fraction bound to a large antigen excess as described by Elliot et al (1987). Typically, mAb were found to contain 50–85% reactive material.

### Binding of [$^{125}$I]mAb to the cell surface

The binding of radiolabelled mAb to cells was determined as described by Elliot et al (1987). Radiolabelled mAb were serially diluted before incubating with cells (0.5–1.25 × 10$^6$ ml$^{-1}$, final volume 1 ml) in RPMI medium containing 10% FCS for 2 h at 37°C. Endocytosis of cell bound mAb was prevented by inclusion of sodium azide (15 mM) and 2-deoxyglucose (50 mM). The cells were separated from the aqueous phase by centrifugation through a 1.1:1 (vol/vol) mixture of dibutyl phthalate:dioctyl phthalate. Radioactivity was measured in a Rackgamma spectrometer (LKB).

### RESULTS

### Sensitivity of Burkitt's lymphoma cell lines to anti-BCR induced growth inhibition

In preliminary experiments, eight Burkitt's lymphoma cell lines, Daudi, Bristol-8, MUTU-I, MUTU-61E, Nalm-6, Namalwa, Ramos and Raji, were assessed for their sensitivity to anti-Fcμ mAb in growth assays. The results obtained with the Ramos, MUTU-I, and Daudi lines with the anti-Fcμ mAb M15/8 are shown in Figure 1. Of the eight lines tested, only Ramos and MUTU-I were sensitive to M15/8, showing a 60–80% inhibition of [$^3$H]thymidine incorporation at a mAb concentration of 10 μg ml$^{-1}$ (Figure 1A, B). Two other anti-Fcμ mAb, Mc41/24G10 and ZL7/5, had similar inhibitory activity on these particular cell lines.

The sensitivity of the eight cell lines to polyclonal anti-μ Ab was also determined. Ramos and Mutu-1 were both highly sensitive to polyclonal anti-μ, giving up to 90% inhibition of [$^3$H]thymidine incorporation at 10 μg ml$^{-1}$ (Figure 1A, B). In addition, the Daudi cell line was sensitive (Figure 1C) which, in the light of its insensitivity to anti-Fcμ mAb, was of particular interest and suggested that the extent of crosslinking of the BCR was probably important in determining growth arrest. The other five cell lines were insensitive to polyclonal anti-μ antibody. Four of the cell lines, Nalm-6, Bristol-8, Raji and Namalwa, expressed relatively low to medium levels of surface IgM compared with Ramos, Daudi, MUTU-I and MUTU-61E (results not shown). Since each of the former group were insensitive to growth arrest, this suggested that although a high level of surface IgM did not guarantee anti-μ mAb-induced inhibition, it was a prerequisite for sensitivity.

© Cancer Research Campaign 1999 

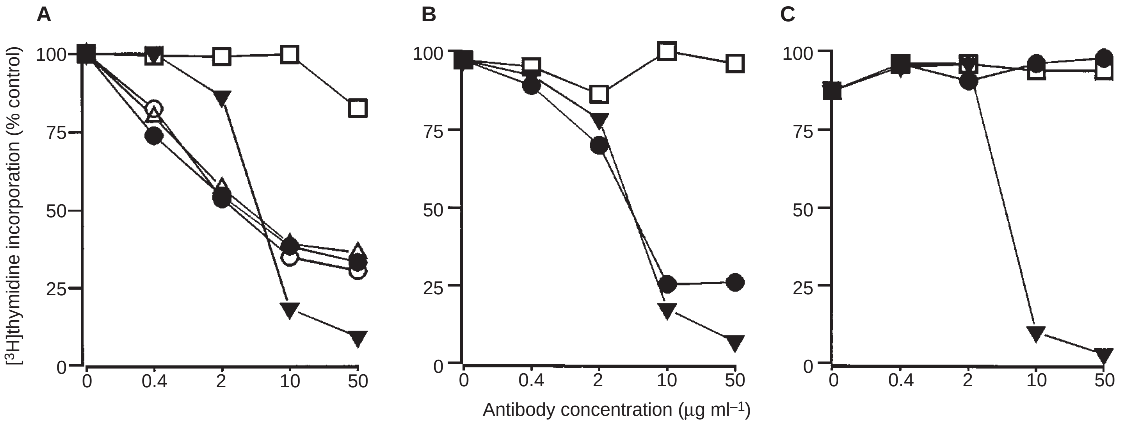

**Figure 1** Growth inhibition of Ramos, MUTU-I, and Daudi cell lines following treatment with monoclonal or polyclonal anti-µ antibody. Ramos (**A**), MUTU-I (**B**), and Daudi (**C**) cells were incubated with antibody at the concentration shown for 24 h, and then with 0.5 µCi [$^3$H]thymidine for 16 h. Cells were then harvested and counted to determine incorporation of radioactivity. The results represent the mean of triplicate determinations. □, irrelevant mAb; ▼, sheep anti-mouse IgG; ●, M15/8 (anti-Fcµ); ○, ZL7/5 (anti-Fcµ); △, Mc41/24G10 (anti-Fcµ). One of at least three similar experiments

**Table 1** The induction of apoptosis in EHRB cells by anti-Fcµ mAb

| Experiment | Treatment | % Apoptotic[d] |
|---|---|---|
| | None | 7 ±1 |
| | Anti-Fcµ[a] | 31 ± 5 |
| Anti-Fcµ multimers | Anti-Fcµ Fab′[b] | 11.5 ± 7 |
| | Anti-Fcµ F(ab′)$_2$[b] | 35 ± 13 |
| | Anti-Fcµ F(ab′)$_3$[b] | 47 ± 5 |
| Hyper-crosslinking | Anti-Fcµ[c] | 12 ± 6 |
| | Anti-Fcµ + sheep anti-mouse IgG[c] | 62 ± 17 |

Cells were incubated with treatment antibody as described below and the level of apoptosis assessed by propridium iodide analysis. The anti-Fcµ mAb used was M15/8. [a,b]Cells were incubated with mAb at 1 µg ml$^{-1}$ for 48 h. [c]Cells were incubated with 10 µg ml$^{-1}$ anti-Fcµ mAb for 15 min, then washed and sheep anti-mouse IgG added for 48 h as described in Figure 5. [d]Values represent mean ± SD for three experiments.

Although initial studies were performed on the Ramos line, several Ramos sub-lines also exist. Two of these, RA.1 (a direct EBV transformant of the original Ramos), and EHRB (a more lymphoblastoid cell type) were assessed for anti-µ mAb-induced growth arrest. While RA.1 cells behaved in a similar manner to the parent line, EHRB cells were found to be particularly sensitive to growth arrest (data not shown) and furthermore succumbed to apoptosis over a period of 24 h in culture with anti-Fcµ mAb (Table 1). EHRB cells were selected for almost all subsequent work. Ramos cells and sublines also have the advantage of being FcR-negative, and so in binding studies whole IgG could be used without interference from Fc:FcR interactions.

### Growth inhibition with pairs of anti-Fcµ mAb

Crossblocking studies showed that the anti-Fcµ mAb M15/8 and ZL7/5 blocked the binding of each other and so bound to identical or overlapping epitopes on the Fc region of IgM (results not shown). The third anti-Fcµ mAb, Mc41/24G10, bound to a distinct epitope on the Fc region since its binding was blocked neither by M15/8 nor by ZL7/5. The effect of both non-crossblocking and crossblocking pairs of anti-Fcµ mAb on DNA synthesis in EHRB and Daudi cells was determined (Figure 2). The results show that on EHRB cells (Figure 2A), the two non-crossblocking pairs of anti-Fcµ mAb (M15/8+Mc41/24G10, and ZL7/5+Mc41/24G10) were markedly more potent inhibitors than the individual mAb or the pair of crossblocking antibodies (M15/8+ZL7/5). Interestingly, using Daudi cells (Figure 2B), which are resistant to inhibition by single anti-Fcµ mAb, pairs of non-crossblocking mAb, like polyclonal anti-µ (Figure 1B), were quite effective and inhibited growth by more than 70%.

Since mAb are directed to a single epitope on the Fcµ region, the most likely explanation for the enhanced response seen with non-crossblocking pairs, is that they crosslink the surface IgM more extensively. This interpretation would be consistent with the potent inhibition of growth seen with polyclonal anti-µ Ab (Figure 1) and suggest that Daudi cells require a higher degree of crosslinking than EHRB in order to generate inhibitory signals.

### Growth inhibition with anti-Fcµ multimers

As an additional method of investigating the role of crosslinking, the performance of Fab′ (univalent), F(ab′)$_2$ (bivalent) and F(ab′)$_3$ (trivalent) prepared from the anti-Fcµ mAb M15/8 were compared on EHRB cells. The results are shown in Figure 3 and demonstrate that increasing mAb valency resulted in increased growth inhibition. Thus Fab′ fragments were ineffective, F(ab′)$_2$ fragments were comparable to the parent IgG, and the F(ab′)$_3$ species was by far the most effective inhibitor. The insert in Figure 3 shows the level of DNA fragmentation achieved as a result of cells entering apoptosis when cultured with the different anti-µ mAb fragments. The trivalent F(ab′)$_3$ induced the highest level of DNA fragmentation. This result was confirmed by measuring the extent of apoptosis in each culture using the method of Nicoletti et al (1991) (Table 1), which showed that the increasing growth inhibition correlated with an increase in the level of apoptosis induced, with the M15/8 Fab′, F(ab′)$_2$ and F(ab′)$_3$ fragments giving 6%, 20% and 40% apoptosis. In addition, several other criteria of apoptosis, including annexin V positivity, chromatin condensation, cell shrinkage and apoptotic body formation, were observed (results not shown).

 © Cancer Research Campaign 1999

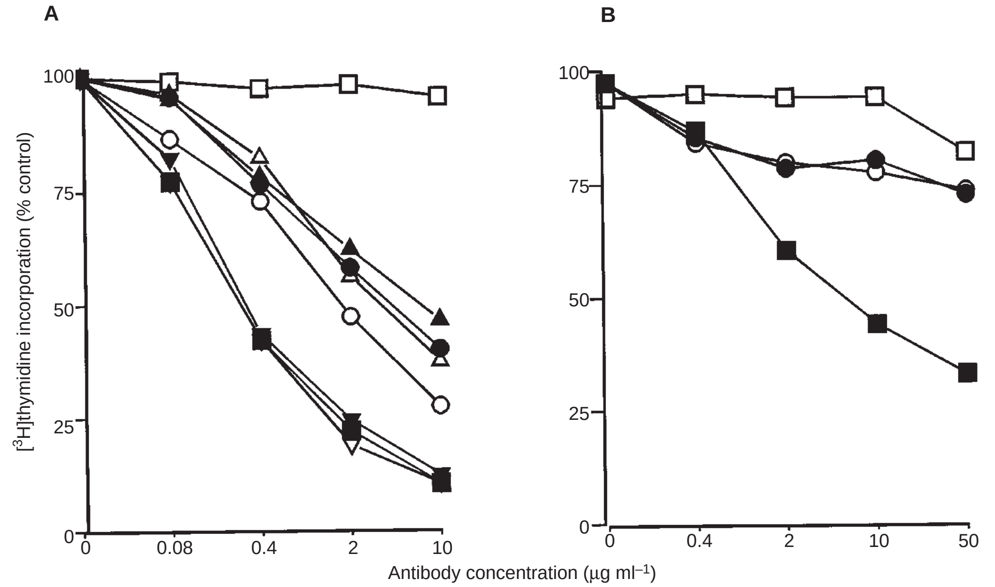

**Figure 2** Growth inhibition of EHRB and Daudi cells with pairs of anti-Fcμ mAbs. EHRB (**A**) and Daudi (**B**) cells were incubated with single or pairs of anti-Fcμ mAb and [3H]thymidine as described in Figure 1. □, irrelevant mAb; ●, M15/8; ○, ZL7/5; △, Mc41/24G10; ▲, M15/8 + ZL7/5; ■, ZL7/5 + Mc41/24G10; ▽, M15/8 + Ms41/24G10; ▼, all three anti-Fcμ mAb. One of at least three similar experiments

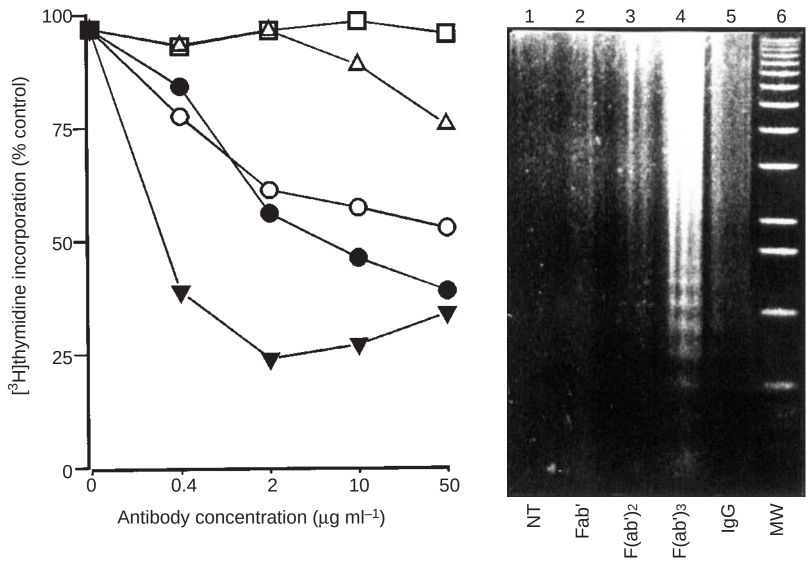

**Figure 3** Comparison of growth inhibition of EHRB cells with anti-Fcμ Fab′, F(ab′)$_2$, and F(ab′)$_3$. Fab′, F(ab′)$_2$, and F(ab′)$_3$ fragments were prepared from M15/8 IgG as described in Materials and methods. Cells were incubated with antibody and [$^3$H]thymidine as described in Figure 1. □, irrelevant mAb; ●, M15/8 IgG; △, M15/8 Fab′; ○, M15/8 F(ab′)$_2$; ▼, M15/8 F(ab′)$_3$. One of at least three similar experiments. *Insert*: Cells incubated with M15/8 Fab′, F(ab′)$_2$, and F(ab′)$_3$ (10 µg ml$^{-1}$) were analysed for DNA fragmentation as described in Materials and methods. Lane 1, untreated; lane 2, Fab′; lane 3, F(ab′)$_2$; lane 4, F(ab′)$_3$; lane 5, IgG; lane 6, molecular weight markers

### Effect of hyper-crosslinking on growth inhibition

As an alternative approach to demonstrate the effect of crosslinking IgM on B-cell growth, we used a layer of polyclonal Ab to extensively crosslink the primary mAb once it had bound to the cell surface. This is termed hyper-crosslinking. The protocol used in these experiments was to allow primary mAb (10 µg ml$^{-1}$) to bind to the cell surface for 15 min, and then to remove the cells from excess mAb by centrifugation before adding the secondary polyclonal anti-mouse IgG Ab. The effect of hyper-crosslinking anti-Fcμ mAb was determined for both EHRB and Daudi cells, and the results are shown in Figure 4. In EHRB cells, hyper-crosslinking was extremely effective at inducing growth arrest (Figure 4A), thus while the anti-Fcμ mAb alone resulted in a maximum of 20% inhibition, in the presence of the hyper-crosslinking Ab it was increased to greater than 90%. This enhanced growth inhibition was associated with an increase in the level of apoptosis from 6% to 43% (Table 1). With Daudi cells

*© Cancer Research Campaign 1999*

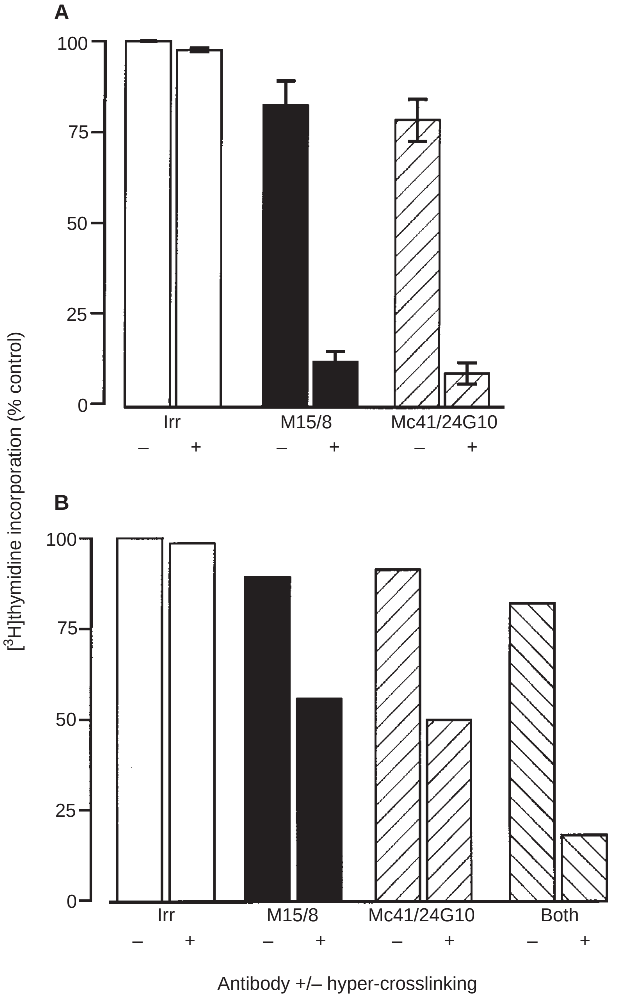

**Figure 4** Growth inhibition of EHRB and Daudi cells following hyper-crosslinking of anti-Fcμ mAb. EHRB (**A**) and Daudi (**B**) cells were incubated with M15/8 (anti-Fcμ) mAb at 10 μg $ml^{-1}$ for 15 min. They were then harvested by centrifugation, resuspended in medium with or without 20 μg $ml^{-1}$ sheep anti mouse IgG, incubated for 24 h at 37°C, and then pulsed overnight with 0.5 μCi/well [$^3$H]thymidine. Cells were harvested and the incorporation of radioactivity determined. The results represent mean ± SD for three experiments for EHRB, and the average of two experiments for Daudi

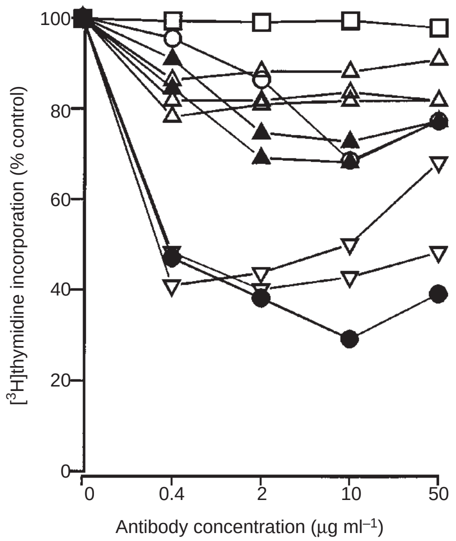

**Figure 5** Comparison of growth inhibition of EHRB cells with mAb recognizing different domains of surface IgM. Cells were incubated with mAb and [$^3$H]thymidine as described in Figure 1. □, irrelevant mAb; ●, M15/8 (anti-Fcμ); ▲, M2E6 and XG9 (anti-Fdμ); ○, Mc24/1C6 (anti-L chain) (M15/2, another anti-L chain mAb gave a similar result); △, ZL16/1, Zl16/3, and ZL16/11 (anti-Ramos Id); ▽, pairs of non-crossblocking anti-Id mAb. One of three similar experiments

(Figure 4B) hyper-crosslinking of a single anti-Fcμ mAb showed less of an inhibitory effect, giving a maximum of 60% inhibition. However, when a pair of non-crossblocking anti-Fcμ mAb was used (M15/8 + Mc41/24G10), the inhibitory effect of hyper-crosslinking was again increased to 90%.

### The effect of domain specificity of anti-IgM mAb on growth inhibition

In the experiments described so far, the mAb used have been directed to epitopes present in the Fc region of the μ-chain (Cμ2–Cμ4 domains). We have previously reported (Zhang et al, 1995) that compared with these, those mAb recognizing epitopes present in the Fab arms of the IgM molecule (anti-Fdμ, anti-L chain, and anti-Id mAb) were comparatively poor in their ability to transduce growth inhibitory signals in Burkitt's cell lines. The results were confirmed and are represented in Figure 5. The two anti-Fdμ mAb, XG9 and M-2E6, reduced growth by up to 25%, compared with 65% for anti-Fcμ mAb. The anti-λ mAb, Mc24/1C6 and M15/2, were also poor at causing growth inhibition as were three mAb specific for the EHRB (Ramos) idiotype, ZL16/1, ZL16/3, and ZL16/11. Crossblocking studies (results not shown) revealed that two of the anti-Id mAb (ZL16/1 and ZL16/3) recognized the same or proximal epitopes on surface IgM, but that the third (ZL16/11) recognized a distinct epitope. In growth arrest assays a mixture of either of the two non-crossblocking pairs, ZL16/1 + ZL16/11 or ZL16/3 + ZL16/11, unlike the single reagents or the crossblocking pair (ZL16/1 + ZL16/3), gave very effective growth inhibition of up to 70%. To determine whether the inhibitory activity of the single anti-Fdμ, anti-L chain or anti-Id mAb could be increased, the effect of hyper-crosslinking these reagents was investigated. The results in Figure 6 show that all types of anti-IgM mAb were highly effective at inducing growth inhibition when hyper-crosslinked by a second layer of polyclonal Ab.

### Stoichiometry and affinity of mAb binding

We next determined the level of binding of $^{125}$I-labelled mAb to EHRB cells; the plots in Figure 7A show the results obtained with representative mAb of each specificity. IgG from the three anti-Fcμ mAb and the two anti-Fdμ mAb gave similar saturation curves and levels of binding, with between 15 and 20 × $10^4$ molecules of mAb binding to each cell. The binding affinities, estimated from these curves, were also similar at around 1 × $10^9$ $M^{-1}$. Surprisingly, however, IgG from three anti-Id mAb and the anti-λ chain mAb gave maximum levels of binding of between 35 and 40 × $10^4$ molecules/cell, approximately twice that obtained with the anti-Fcμ and anti-Fdμ mAb. Similar binding stoichiometry was obtained with anti-Fcμ and anti-Id mAb on Daudi cells

 © Cancer Research Campaign 1999

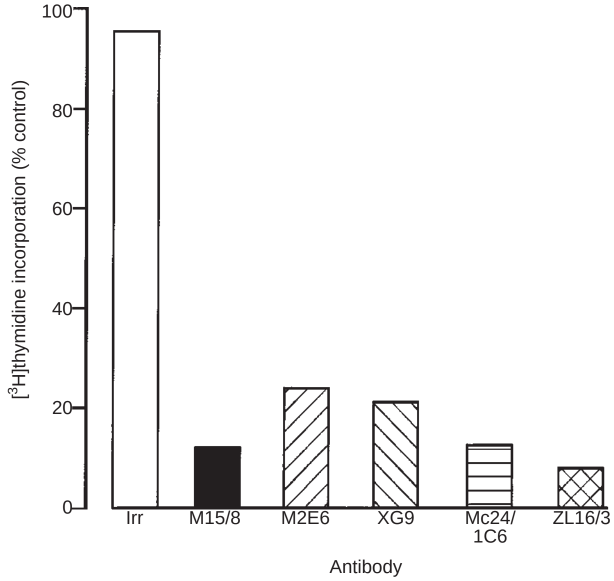

**Figure 6** Growth inhibition of EHRB cells following hyper-crosslinking of mAb recognizing different domains on surface IgM. Cells were incubated with mAb followed by sheep anti-mouse IgG as described in Figure 4

(anti-Id mAb raised to Daudi IgM) and with anti-Fcμ and anti-L chain mAb on Namalwa cells (results not shown): i.e. anti-Id and anti-L chain mAb bound approximately twice as many molecules of mAb per cell as anti-Fcμ mAb.

In view of these somewhat unexpected findings, we next compared the binding of radiolabelled $F(ab')_2$ and Fab′ fragments from the anti-Fcμ mAb M15/8 (Figure 7B) and the anti-L chain mAb Mc24/1C6 (Figure 7C). The results are represented as Scatchard plots in Figure 7B and C. The binding pattern obtained with the anti-Fcμ mAb preparations (Figure 7B) were as expected, with the $F(ab')_2$ behaving very like the parent IgG with regard to affinity of binding and the number of molecules bound to each cell, and with the Fab′ fragments having a lower affinity and binding approximately twice as many molecules to each cell. These results are consistent with two molecules of univalent Fab′ binding to each molecule of surface IgM and one molecule of bivalent $F(ab')_2$ binding per surface IgM. In contrast, the affinity and maximum level of binding obtained with $F(ab')_2$ and Fab′ from the anti-L chain mAb (Figure 7C) were both surprisingly similar and close to the value obtained with the parent IgG. Thus these results are consistent with the Mc24/1C6 IgG and $F(ab')_2$ fragments binding univalently and so giving a 2:1 stoichiometry of $F(ab')_2$:surface IgM.

### DISCUSSION

In this work we have investigated the ability of various anti-IgM mAb to induce growth arrest and apoptosis of a number of Burkitt's cell lines. The preliminary screening showed that of eight lines tested, only two, Ramos and Mutu-1, were sensitive to growth inhibition by anti-Fcμ mAb, and that Daudi cells, although not sensitive to anti-Fcμ mAb, showed inhibition in response to polyclonal anti-IgM. The differential sensitivity of the lines to anti-IgM inhibition probably relates to a number of factors. George et al (1993) have demonstrated that surface receptor number and density are important criteria in B-cell signalling. Our results showing that anti-μ Ab, whether polyclonal or monoclonal, was unable to induce growth inhibition in any of the low/medium IgM-expressing cell lines tested (Namalwa, Raji, Bristol-8 and Nalm-6), support this observation. This is in contrast to the reports from Rudich et al (1988) and Mongini et al (1992) who demonstrated that BCR-induced growth inhibition is much less dependent on receptor density than is B-cell activation. The insensitivity of some of the lines may also be related to their differentiation stage, as it is known that BCR ligation only signals for inhibition at certain stages during B-cell development, and may reflect the levels of anti- and pro-apoptotic proteins present. The EBV status of the cells (Gregory et al, 1991) and whether or not they possess FcR may also influence their sensitivity to growth inhibition and apoptosis. The Ramos EHRB sub-line selected for the main part of the project was exquisitely sensitive to anti-IgM-mAb-induced growth arrest and had the advantage that it did not express FcR, and so the inhibitory response could not be influenced by FcR:Fc interactions.

The two principle findings of the study were: 1) that the more surface IgM was crosslinked by mAb, the more effective was the

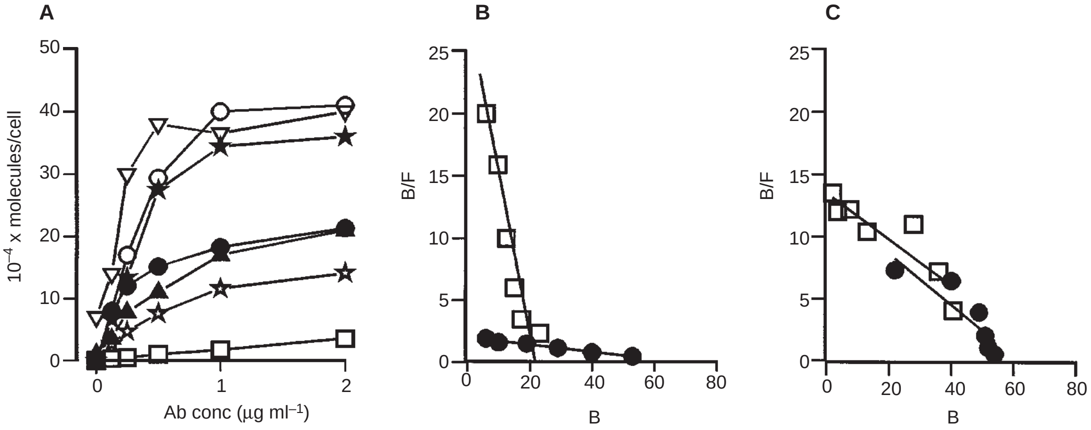

**Figure 7** Binding of [$^{125}$I]-labelled mAb to EHRB cells. (**A**) Serial dilutions of [$^{125}$I]-labelled mAb were incubated with EHRB cells (1–2.5 × 10$^6$) for 2 h at 37°C. The cells were then sedimented through phthalate oils and the pellet counted for radioactivity. The results are expressed as the number of antibody molecules bound per cell. □, irrelevant mAb; ●, M15/8 (anti-Fcμ); ▲, XG9, and ☆, M2E6 (anti-Fdμ); ▽, ZL16/1, and ★, ZL16/3 (both anti-Ramos Id); ○, Mc24/1C6 (anti-L chain). One of at least three similar experiments. (**B, C**) Scatchard plots to compare the binding of $F(ab')_2$ (□) and Fab′ (●) from M15/8 (**B**) and Mc24/1C6 (**C**) to EHRB cells. B, molecules bound per cell (× 10$^{-4}$); F, free antibody concentration (M)

© Cancer Research Campaign 1999

resultant growth arrest and apoptosis; and 2) that the ability of a single mAb to induce crosslinking was highly dependent on epitope specificity. A range of experiments using anti-IgM mAb (single or cocktails), polyclonal anti-μ Ab, and hyper-crosslinking, all showed that growth arrest of B-cell lines was highly dependent on the extent of IgM crosslinking. These observations were confirmed by varying the valency of the mAb by using Fab′, $F(ab')_2$ and $F(ab')_3$ derivatives from anti-Fcμ mAb. Here the progressive increase in valency resulted in a corresponding decrease in cell proliferation, presumably as a result of more efficient BCR-crosslinking on the cell surface. Furthermore, we were able to show that cells treated with a trivalent $F(ab')_3$ anti-μ derivative underwent extensive DNA fragmentation indicating induction of apoptosis (insert to Figure 3).

Interestingly, the ability of single mAb to mediate IgM crosslinking activity was dependent on their epitope specificity. Thus, mAb directed at the Fc region (Cμ2–Cμ4 domains) of surface IgM were much more effective at crosslinking B-cell IgM and inducing growth arrest, than were those directed at Fab region (anti-L chain, anti-Id and anti-Fdμ) of this molecule. Only when these latter mAb were hyper-crosslinked with polyclonal anti-mouse IgG did they cause appreciable growth arrest and apoptosis. Binding studies showed that this difference in function was not related to any marked disparity in the affinity of the mAb studied which were all in the range $1–4 \times 10^9$ $M^{-1}$ (Figure 6). In view of the results showing the importance of crosslinking in achieving growth inhibition, this probably relates to the intrinsic crosslinking capacity of the mAb, and depends on whether the anti-Fc and anti-Fab mAb bind to surface IgM bigamously or monogamously (Elliot et al, 1987). mAb that bind bigamously, with epitopes or adjacent BCR complexes, form effective inter-BCR crosslinks, and those that bind monogamously, with the epitopes on a single surface IgM molecule, have poor crosslinking capacity. Rudich et al (1988) have provided evidence using immunoelectron microscopy that whereas the anti-Fdμ mAb XG9, used in this study, binds in a predominantly monogamous manner, an anti-Fcμ mAb, 1G6, binds predominantly bigamously. This could explain the inefficacy of XG9 in our assays and supports the hypothesis that bigamous binding is a requirement for BCR-induced growth inhibition. Grafton et al (1997) have recently published a study using a large panel of anti-IgM mAb recognizing the Cμ2–Cμ4 domains of the Fc region. They show that most of these mAb were able to induce growth inhibition; interestingly, although the panel did not include any mAb recognizing the anti-Fdμ region (Cμl domain), two anti-Id mAb in the panel failed to cause growth inhibition.

Although initially the lack of efficacy of the anti-L chain and anti-Id mAb was also attributed to a predominantly monogamous type binding, the results of the $^{125}$I-labelled mAb binding experiments suggested an alternative explanation (Figure 7). The maximum level of binding to EHRB cells obtained with IgG from the anti-L chain and anti-Id mAb was twice that obtained with the anti-Fcμ and anti-Fdμ mAb. Similar results were obtained with anti-Id mAb on Daudi cells and anti-L chain mAb on Namalwa cells. Assuming that the stoichiometry of anti-Fdμ and anti-Fcμ mAb binding to surface IgM molecule is 1:1, this suggests that two molecules of anti-L chain or anti-Id mAb were bound per surface IgM, and therefore favours the idea that their binding was predominantly univalent rather than bivalent, and that this is the reason for their poor growth inhibitory capacity. The comparison of the binding of $F(ab')_2$ and Fab′ fragments (Figure 7) was consistent with this interpretation, since, in contrast to the results obtained with fragments from the anti-Fcμ mAb M15/8, Fab′ and $F(ab')_2$ from anti-L chain mAb gave a similar value for maximum binding and binding affinity.

In view of the therapeutic success of anti-Id mAb in certain B-cell malignancies (Vuist et al, 1994; Racila et al, 1995; Tutt et al, 1998), the inability of any of the three anti-Ramos Id mAb (and two for Daudi) to cause growth inhibition in vitro was somewhat surprising. This is particularly important because there is growing support for the idea that a number of therapeutic anti-lymphoma mAb, including anti-Id and anti-CD20, may be operating via a direct signalling route, rather than by the recruitment of cytotoxic effector cells (Tutt et al, 1998). However, our results showed that irrespective of the ability of an individual mAb to induce growth inhibition, after hyper-crosslinking all mAb were equally potent. The ability of anti-Id mAb to induce transmembrane signalling and growth arrest in vivo is probably dependent on a number of factors other than their intrinsic crosslinking activity, which we have measured. For example, we can expect that FcR-bearing effector cells might play an important role in delivering hyper-crosslinking to anti-Id mAb-coated target cells. In this situation many FcR-carrying cells such as NK-cells, monocytes, neutrophils, endothelial cells or perhaps even B-cells would be effective at delivering cytotoxic signal. Thus effector cells would not necessarily need to be cytotoxic in themselves, but would simply need to deliver a multivalent array of FcR to hyper-crosslink the BCR. In support of this suggestion, recent work from Shan et al (1998) shows that the ability of another apoptosis-inducing mAb, anti-CD20, is markedly enhanced by the addition of FcR-bearing effector cells to the cultures. The presence of FcR-bearing cells has also been shown to enable a mAb recognizing CD79β, a signalling component of the BCR, to induce inhibitory signals (Van Kooten et al, 1997). The results presented here show that in vitro most anti-Id mAb are particularly ineffective at crosslinking the BCR to mediate growth arrest, suggesting that their success in vivo probably require an additional form of crosslinking.

### ACKNOWLEDGEMENTS

We would like to thank Dr A Milner and Prof Chris Gregory (Birmingham) and Dr Bill Cushley (Glasgow) for cell lines, and Dr P Mongini (New York) for antibody. This work was supported by Tenovus of Cardiff, the European Union, and the Leukaemia Research Fund.

### REFERENCES

Bonardi MA, French RR, Amlot P, Gromo G, Modena D and Glennie MJ (1993) Delivery of saporin to human B-cell lymphoma using bispecific antibody targeting via CD22 but not CD19, CD37 or immunoglobulin results in efficient killing. *Cancer Res* **53**: 3015–3021

De Franco AL, Raveche ES, Asofsky R and Paul WE (1982) Frequency of lymphocytes b responsive to anti-immunoglobulin. *J Exp Med* **155**: 1523–1536

Elliot TJ, Glennie MJ, McBride HM and Stevenson GT (1987) Analysis of the interaction of antibodies with immunoglobulin idiotype of neoplastic B lymphocytes; implications for immunotherapy. *J Immunol* **138**: 981–988

George J, Penner SJ, Weber J, Berry J, Caflin JL (1993) Influence of membrane Ig receptor density and affinity on B cell signaling by antigen. *J Immunol* **151**: 5955–5965

Glennie MJ, Tutt AL and Greenman J (1993) Preparation of multispecific $F(ab')_2$ and $F(ab')_3$ antibody derivatives. In *Tumour Immunobiology, a Practical Approach*, Rees RC, Reynolds CW (eds), pp. 225–244. Oxford University Press: Oxford

© Cancer Research Campaign 1999

Gold MR, Matsuuchi L, Kelly RB and De Franco AL (1991) Tyrosine phosphorylation of components of the B-cell antigen receptors following receptor crosslinking. *Proc Natl Acad Sci USA* **88**: 3436–3440

Grafton G, Goodall M, Gregory CD and Gordon J (1997) Mechanisms of antigen receptor-dependent apoptosis of human B lymphoma cells probed with a panel of 27 monclonal antibodies. *Cell Immunol* **182**: 45–56

Gregory CD, Dive C, Henderson S, Smith CA and Williams GT (1991) Activation of EBV latent genes protects human B cells from death by apoptosis. *Nature* **349**: 612–614

Gregory CD, Rowe M and Rickinson AB (1990) Different EBV-B cell interactions in phenotypically distinct clones of a Burkitt's lymphoma cell line. *J Gen Virol* **71**: 1481–1495

Hasbold J and Klaus GGB (1990) Anti-immunoglobulin antibodies induce apoptosis in immature B-cell lymphomas. *Eur J Immunol* **20**: 1685–1690

Marches R, Racila E, Tucker TF, Picker L, Mongini P, Hsueh R, Vitetta ES, Scheuermann RH and Uhr JW (1995) Tumour dormancy and cell signalling – III: role of hypercrosslinking of IgM and CD40 on the induction of cell cycle arrest and apoptosis in B lymphoma cells. *Therap Immunol* **2**: 125–136

Nicoletti I, Migliorati G, Pagliacci MC, Grignani F and Riccardi C (1991) A rapid and simple method for measuring thymocyte apoptosis by propridium iodide staining and flow cytometry. *J Immunol Methods* **139**: 271–279

Racila E, Schuermann RH, Picker LJ, Yefenof E, Tucker T, Chang W, Marches R, Street NE, Vitetta ES and Uhr JW (1995) Tumor dormancy and signalling, II. Antibody as an agonist in inducing dormancy of a B cell lymphoma in SCID mice. *J Exp Med* **181**: 1539–1550

Rudich SM, Roux KH, Winchester RJ and Mongini PKA (1988) Anti-IgM B cell mediated signaling – molecular analysis of ligand binding requisites for human B cell clonal expansion and tolerance. *J Exp Med* **168**: 247–266

Sefton BM and Campbell MA (1991) The role of tyrosine phosphorylation in lymphocyte activation. *Ann Rev Cell Biol* **7**: 257–274

Shan, D, Ledbetter JA and Press OW (1998) Apoptosis of malignant human B cells by ligation of CD20 with monoclonal antibodies. *Blood* **91**: 1644–1652

Tutt AL, Stevenson GT and Glennie MJ (1991) Trispecific $F(ab)_3$ derivatives that use cooperative signalling via the TCR/CD3 complex and CD2 to activate and redirect resting cytotoxic T cells. *J Immunol* **147**: 60–69

Tutt AL, French RR, Illidge TM, Honeychurch J, McBride H, Penfold C, Fearon DT, Parkhouse ME, Klaus GGB and Glennie MJ (1998) Monoclonal antibody therapy of B-cell lymphoma: signaling activity on tumour cells appears more important than recruitment of effectors. *J Immunol* (in press)

Van Kooten, Galiber L, Seon BK, Garrone P, Liu Y-J and Banchereau J (1997) Cross-linking of antigen receptor via Ig-β (B29, CD79b) can induce both positive and negative signals in CD40 activated human B cells. *Clin Exp Immunol* **110**: 509–515

Vuist WMJ, Levy R and Maloney DG (1994) Lymphoma regression induced by monoclonal anti-idiotype antibodies correlates with their ability to induce Ig signal transduction and is not prevented by tumour expression of high levels of bcl-2 protein. *Blood* **83**: 899–906

Zhang I, French RR, Chan HTC, Cragg MS, Power MJD and Glennie MJ (1995) The development of anti-CD79 monoclonal antibodies for treatment of B-cell neoplastic disease. *Therap Immunol* **2**: 191–202

*© Cancer Research Campaign 1999*
